# Recovered COVID-19 patients with recurrent viral RNA exhibit lower levels of anti-RBD antibodies

**DOI:** 10.1038/s41423-020-00528-0

**Published:** 2020-09-16

**Authors:** Bingfeng Liu, Yaling Shi, Wanying Zhang, Rong Li, Zhangping He, Xiaofan Yang, Yuejun Pan, Xilong Deng, Mingkai Tan, Lingzhai Zhao, Fan Zou, Yiwen Zhang, Ting Pan, Junsong Zhang, Xu Zhang, Fei Xiao, Fang Li, Kai Deng, Hui Zhang

**Affiliations:** 1 Institute of Human Virology, Key Laboratory of Tropical Disease Control of Ministry of Education, Guangdong Engineering Research Center for Antimicrobial Agent and Immunotechnology, Zhongshan School of Medicine, Sun Yat-sen University, Guangzhou, Guangdong China; 2 Guangzhou Eighth People’s Hospital, Guangzhou Medical University, Guangzhou, Guangdong China; 3 Qianyang Biomedical Research Institute, Guangzhou, Guangdong China; 4 Institute of Pediatrics Guangzhou Women and Children Hospital, Guangzhou Medical University, Guangzhou, Guangdong China; 5 Fifth Affiliated Hospital, Sun Yat-sen University, Zhuhai, Guangdong China

**Keywords:** Predictive markers, Infectious diseases

Coronavirus disease 2019 (COVID-19), caused by infection with severe acute respiratory syndrome coronavirus 2 (SARS-CoV-2), has rapidly become a global pandemic. Most discharge criteria for patients with COVID-19 recommend two consecutive negative reverse-transcriptase polymerase-chain reaction (RT-PCR) test results from respiratory specimens over a 24-hour interval. Reports are accumulating of recurrent viral RNA-positive (RP) nasopharyngeal or anal specimens from recovered patients in China, Italy, South Korea, and France.^1–8^ Two cohort studies reported that 14.5% (38/262) and 16.7% (69/414) of patients with COVID-19 retesting were positive for SARS-CoV-2 RNA after discharge.^2,6^ The potential causes of RP specimens may be related to several aspects, such as virological factors, including residual viral reservoir, intermittent viral release, tissue distribution or false negatives, as well as immunological and sampling methodological factors.^3,9,10^ Given that an increasing number of RP cases have been reported worldwide, this phenomenon cannot be simply ascribed to false-negative testing. It indeed occurs, as truly discharged patients could suffer reactivation or could be reinfected with SARS-CoV-2.^3^ The risk of possible retransmission still exists, and the serological characteristics remain largely unknown, which could jeopardize the management of recovered COVID-19 patients.^11^

In this study, we collected blood samples from 47 recovered patients. The median age of the patients was 49 years (range: 17–82 years), and 55.3% of them were women. According to the Chinese national treatment guidelines, their symptoms were classified as moderate (36 patients) or severe (11 patients), and the median disease duration (from the onset of illness to the last RNA-negative conversion before RP status) was 15 days (range: 1–40 days). Eight of the 47 recovered patients displayed RP anal or throat swab samples from 8 to 39 days after viral shedding, and 39 patients remained persistently RNA negative (PRN). None of the conditions of these RP patients were severe before recovery (Fig. S1a). The median disease duration was 16.5 days for RP patients (ranging from 10 to 24 days), which was not significantly different from that of PRN patients (15 days, ranging from 1 to 40 days) (Fig. S1b). Moreover, the RP patients were significantly younger than the PRN patients (median 34.5 years vs. 54 years, *p* = 0.019), which is consistent with previous reports (Fig. S1c).^1,2^

Plasma samples were collected from these 47 patients with COVID-19 at the time of convalescence and assessed for antibodies against the following SARS-CoV-2 proteins: the spike glycoprotein (S); the receptor-binding domain (RBD); conserved heptad repeats (HR1–HR2) in the S2 domain; and the nucleocapsid (N), membrane (M), and envelope (E) proteins. The concentrations of IgG secreted in response to these SARS-CoV-2 proteins varied in different patients, with detection rates of 100.0% (47/47), 83.0% (39/47), 97.9% (46/47), 100.0% (47/47), 55.3% (26/47), and 21.3% (10/47) for the S, RBD, HR1–HR2, N, M, and E proteins, respectively (Fig. 1a; Fig. S2). The detection rates of IgM to the S, RBD, HR1–HR2, and N proteins were 100.0% (47/47), 95.7% (45/47), 83.0% (39/47), and 100% (47/47), respectively (Fig. 1b; Fig. S2). Notably, significantly higher levels of SARS-CoV-2-specific IgG and IgM developed to the S and N proteins (Fig. 1a, b).

**Fig. 1:**
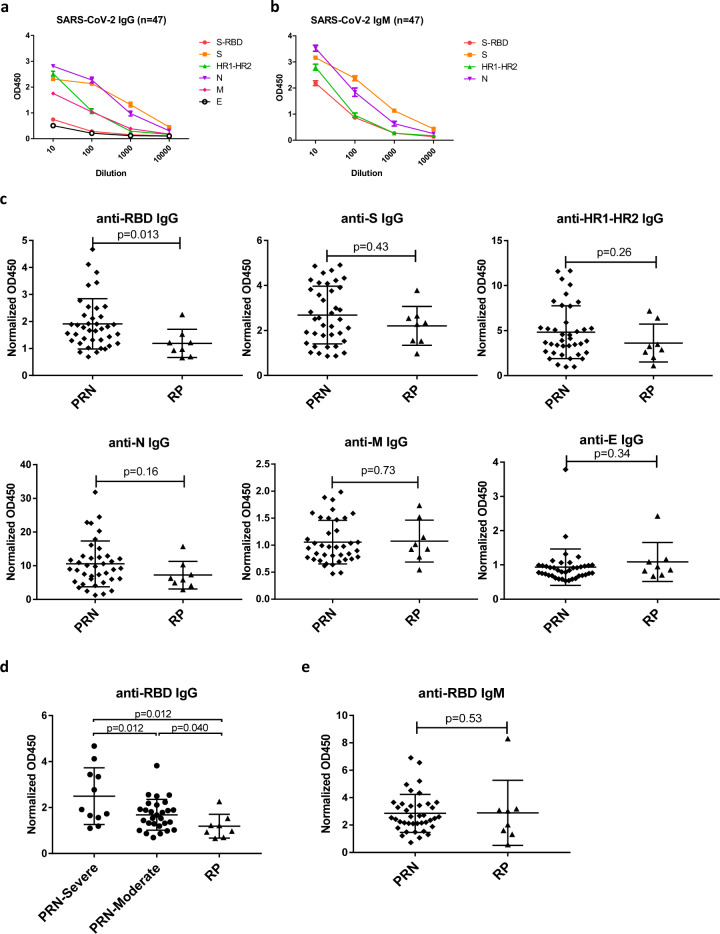
Comparison of antibody responses to SARS-CoV-2 between PRN and RP patients. Kinetics of binding IgG (**a**) and IgM (**b**) targeting the RBD, S, HR1–HR2, N, M, and E proteins in 47 recovered COVID-19-patient plasma samples collected during the convalescent period are shown. The *Y*-axis represents optical density units at OD450 nm, and the *X*-axis represents reciprocal plasma dilutions. **c** Normalized OD450 nm values of the anti-SARS-CoV-2 IgG to the RBD, S, HR1–HR2, N, M, and E proteins are compared between PRN and RP patients. The *P* value was calculated using a two-tailed Mann–Whitney *U* test or unpaired Student’s *t* test. **d** Normalized OD450 nm values of the anti-RBD IgG were compared between PRN-severe, PRN-moderate, and RP patients. The *P* value was calculated using a two-tailed Mann–Whitney *U* test or unpaired Student’s *t* test. **e** Normalized OD450 nm values of the anti-RBD IgM were compared between PRN and RP patients. The *P* value was calculated using a two-tailed Mann–Whitney *U* test

To evaluate the effect of specific antibodies on RP status, we compared the levels of anti-SARS-CoV-2 IgG to the S, RBD, HR1–HR2, N, and M proteins in these patients during their convalescent period (Fig. 1c; Fig. S3). The results showed that RP patients induced significantly lower levels of anti-RBD IgG than PRN patients (*p* = 0.013) (Fig. 1c). As all of these RP patients were in a moderate condition before recovery, the PRN patients were further classified as moderate (28 patients) or severe (11 patients) according to their symptoms before recovery. The levels of anti-RBD IgG in RP patients were still significantly lower than those of either PRN-severe or PRN-moderate patients (*p* = 0.012 and *p* = 0.040, respectively; Fig. 1d). In addition, the patients with severe symptoms within the PRN group were more likely to induce higher levels of anti-RBD IgG (*p* = 0.012; Fig. 1d), which is consistent with previous reports.^12^ In contrast, there were no significant differences either in IgG to other viral proteins or in IgM between PRN and RP patients (Fig. 1c, e; Figs. S4, S5), suggesting that the humoral response to RBD rather than to other regions of the S protein or the full-length S protein might have played an important role in preventing viral rebound during recovery.

Furthermore, we observed that the titers of IgG to RBD among these recovered patients positively correlated with the spike-binding antibodies targeting the S, HR1–HR2, and N proteins (*r* = 0.71, *p* < 0.0001; *r* = 0.53, *p* < 0.0001; and *r* = 0.33, *p* = 0.022, respectively) but not with the M or E proteins (Fig. S6a). Moreover, the level of IgM to the RBD protein among these recovered patients also correlated with the S, HR1–HR2, and N proteins (*r* = 0.67, *p* < 0.0001; *r* = 0.56, *p* < 0.0001; and *r* = 0.60, *p* < 0.0001, respectively) (Fig. S6b). In addition, a positive correlation was also observed between age and IgG level to the RBD, S, HR1–HR2, and N proteins (*r* = 0.38, *P* = 0.0077; *r* = 0.40, *P* = 0.0055; *r* = 0.45, *P* = 0.0017; and *r* = 0.44, *P* = 0.0021, respectively; Fig. S7), indicating the important role of age in the generation of specific binding antibodies.^13^

Because of the lack of clinical characteristics and the unknown significance of RP patients, it is critical to provide comprehensive serological profiling to guide the management of recovered COVID-19 patients after discharge. An important feature of the RP patients was their younger age than that of the PRN patients, and the ages of these recovered patients positively correlated with titers of IgG to the RBD protein.^1,2,13^ These observations are consistent with the conclusion that the level of IgG to the RBD protein in RP patients is significantly lower than that in the PRN group.

Based on our findings, the anti-RBD IgG level could serve as an indicator of RP status. To minimize the risk of possible viral rebound and retransmission during the current pandemic, close monitoring of anti-RBD IgG levels at viral shedding and a long-term follow-up of patients with lower levels of RBD antibodies is needed. Moreover, the relationship between anti-SARS-CoV-2 IgG titers and RP status suggests that the interplay between the virus and the host immune response in coronavirus infections should be further investigated for the development of more accurate diagnostic technologies and effective vaccines against viral infection.

## Supplementary information

Materials and Methods, Supplementary Figures S1-S7
