# Supplementary material for: Recovered COVID-19 patients with recurrent viral RNA exhibit lower levels of anti-RBD antibodies
Source: Cell Mol Immunol. 2020 Sep 16;17(10):1098–100. doi: 10.1038/s41423-020-00528-0 (PMC7493297; doi:10.1038/s41423-020-00528-0)
Supplement: Supplementary file 1 — Materials and Methods, Supplementary Figures S1-S7 [file 41423_2020_528_MOESM1_ESM.docx]

**Additional information**

This appendix has been provided by the authors to give readers additional information about their work.

**Recovered COVID-19 Patients with Recurrent Viral RNA Positive Exhibit Lower Anti-RBD Antibodies**

Bingfeng Liu, Yaling Shi, Wanying Zhang, Rong Li, Zhangping He, Xiaofan Yang, Yuejun Pan, Xilong Deng, Mingkai Tan, Lingzhai Zhao, Fan Zou, Yiwen Zhang, Ting Pan, Junsong Zhang, Xu Zhang, Fei Xiao, Fang Li, Kai Deng and Hui Zhang

**Table of Contents**

[**Materials and Methods** 2](#_Toc47277228)

[**Supplementary Figure** 5](#_Toc47277229)

**Materials and Methods**

**Study Oversight**

The study included a cohort of 47 COVID-19 patients admitted to Guangzhou Eighth People’s Hospital or the Fifth Affiliated Hospital of Sun Yat-sen University, and the study was approved by the internal review board (IRB) of both hospitals. All patients were diagnosed as COVID-19 with laboratory-confirmed positive respiratory RT-PCR tests. They were discharged after meeting the requirement of effective national treatment standards and additional two-week quarantine. The discharge criteria of the recovered patients included: haemodynamically stable and afebrile for longer than 3 days, respiratory symptoms significantly improved, and significant absorption of pulmonary lesions of chest CT imaging, and at least consecutive negative RNA test results for two apart from each other by over a 24-hour interval on nasopharyngeal and oropharyngeal swabs, and no concurrent acute medical issues requiring transfer to another medical facility. All recovered patients were followed-up for at least two additional weeks after viral shedding. During this follow-up, the RP patients were confirmed by 3 repeat re-detected positive of RT-PCR tests in nasopharyngeal and anal specimens. An additional RT-PCR test was performed using a kit from a different manufacturer and the results were also positive for all patients. The RP patients re-admitted to hospital for further medical observation and close contacts were also followed-up.

**ELISA**

The recombinant His-tagged N of SARS-CoV-2 was expressed in *E. coli* by pET28a expression system. RBD (amino acids 318-540), HR1-HR2 (amino acids 892-1195) and M of SARS-CoV-2 with His-tag were expressed by a HEK293FT cells. The His-tagged proteins were applied to a Ni-conjugated agarose bead column (GE) for purification. Then the proteins were suspended in PBS buffer and the concentration was measured by the Bradford method. Full length His-tagged S (40592-V08H) was purchased from Sino Biological Inc and His-tagged E (DRA33) was purchased from Novoprotein.

SARS-CoV-2 S, RBD, HR1-HR2, N, M, or E proteins (5 μg/ml) were coated on a Costar Stripwell^TM^ Microplate at 4 °C overnight (50 μl/well). The plates were blocked with 5% non-fat milk in PBS for two hours at 37 °C, subsequently were washed three times with PBS containing 0.1% v/v Tween-20 (PBS-T), followed by incubation with serial 10-fold diluted plasma from recovered COVID-19 patients in PBS-T for 1 hour at 37 °C (Plasma from healthy donors was used as a negative control). After three washes with PBS-T, A 1:10000 dilution of horseradish peroxidase (HRP)-conjugated goat anti-human IgG antibody (SSA002-1, Sino Biological Inc) or HRP-conjugated goat anti-human IgM antibody (A18841, Invitrogen) was added for 1 hour at 37 °C. The plates were then washed four times with PBS-T, followed by adding 100 μl of tetramethylbenzidine (TMB) substrate (Invitrogen) at room temperature in the dark. After 10 minutes, the reaction was stopped with 100 μl 2 M H_2_SO_4_ solution. The absorbance was measured at 450 nm.

**Statistical analysis**

A statistical analysis was performed using PRISM version 7.0. All of the statistical tests were two tailed, and significant differences were considered at p < 0.05. Continuous variables were evaluated using Mann-Whitney U test or unpaired t-test. Spearman’s correlation to assess the relationships between continuous variables.

**Study approval**

The study was performed in accordance with Good Clinical Practice and the Declaration of Helsinki principles for ethical research. The study protocol was approved by the Institutional Review Board of Guangzhou Eighth People’s Hospital (Guangzhou, China) and the Fifth Affiliated Hospital of Sun Yat-sen University (Zhuhai, China). All patients provided written informed consent for their participation in the study.

**Supplementary Figure**

**
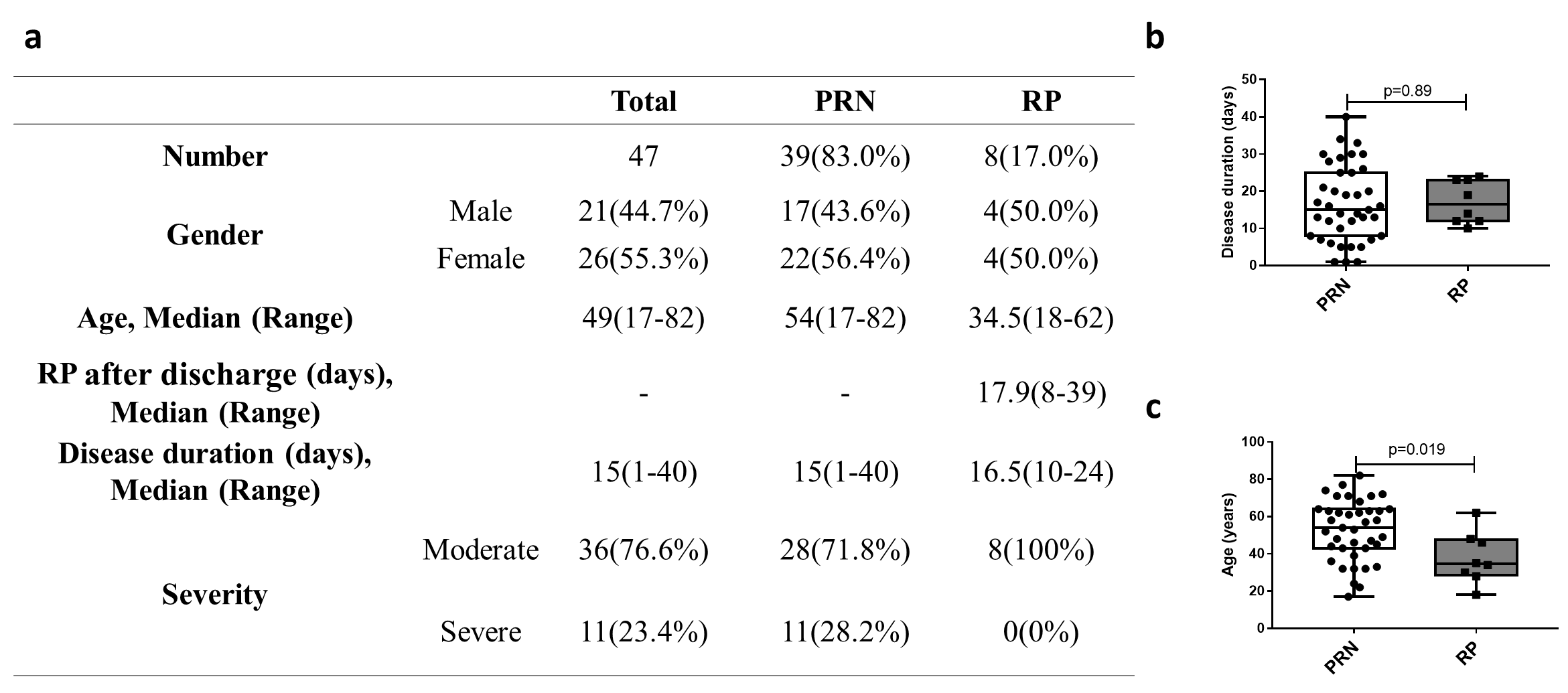
**

**Supplementary Figure S1. Demographics and disease durations of study participants. a** Characteristics of enrolled patients with COVID-19. Disease durations (**b**) and ages (**c**) of recovered patients are compared between PRN and RP patients. P value was calculated using two tailed unpaired Student’s t-test.


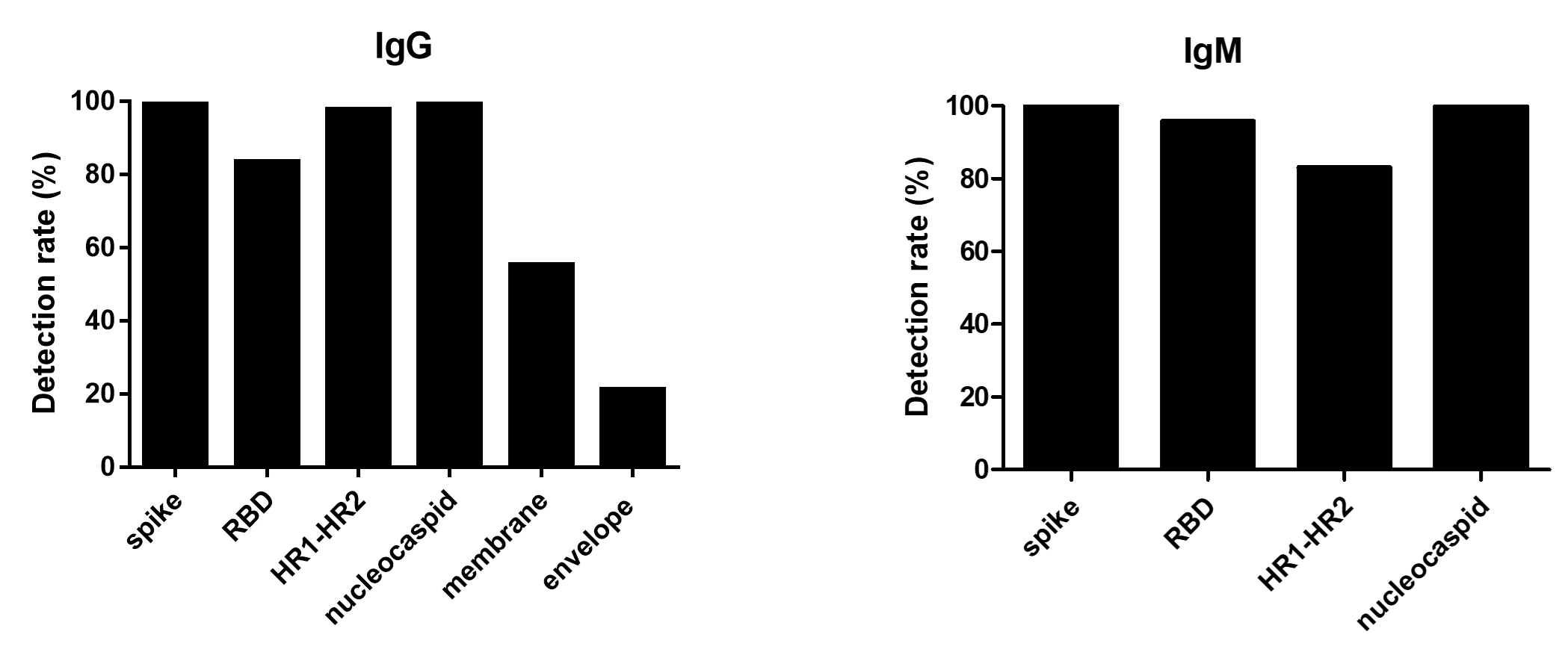


**Supplementary Figure S2.** The detection rates of IgG (left panel) and IgM (right panel) to SARS-CoV-2 proteins in 47 COVID-19 patient plasma at the convalescent period are shown.


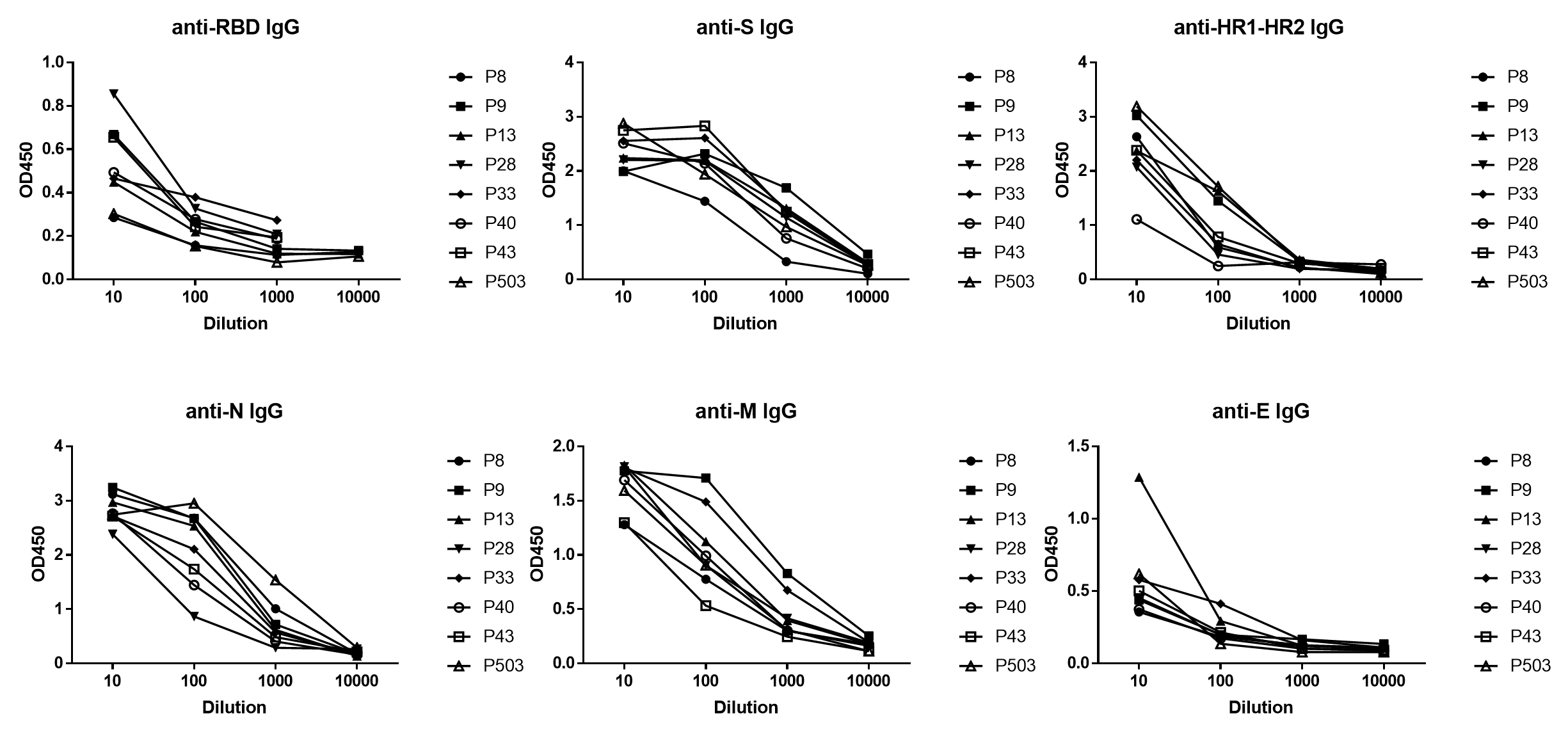


**Supplementary Figure S3.** Kinetics of binding IgG targeting RBD, S, HR1-HR2, N, M and E protein in 8 RP patient plasma at the convalescent period are shown.


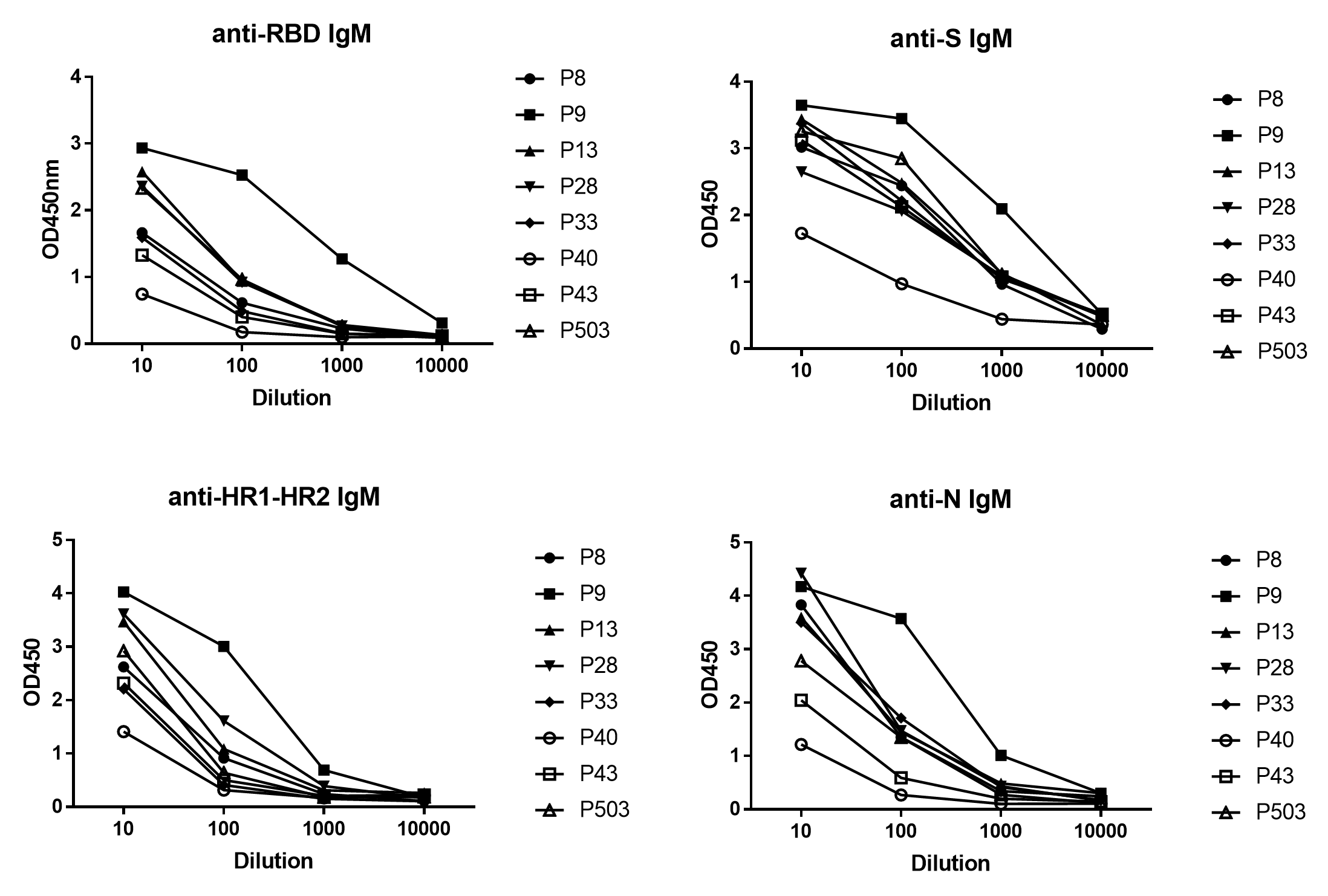


**Supplementary Figure S4.** Kinetics of binding IgM targeting RBD, S, HR1-HR2 and N protein in 8 RP patient plasma at the convalescent period are shown.


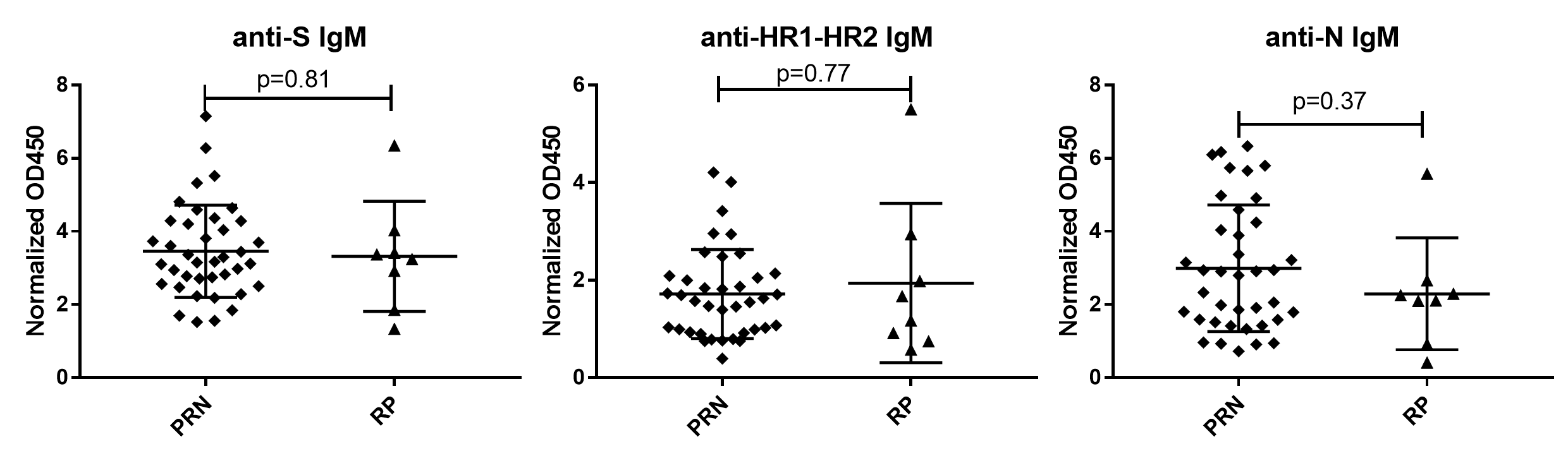


**Supplementary Figure S5.** Normalized OD450 nm values of the anti-SARS-CoV-2 IgM to S, HR1-HR2 and N are compared between NRP and RP patients. P value was calculated using two tailed unpaired Student’s t-test or Mann-Whitney U test.


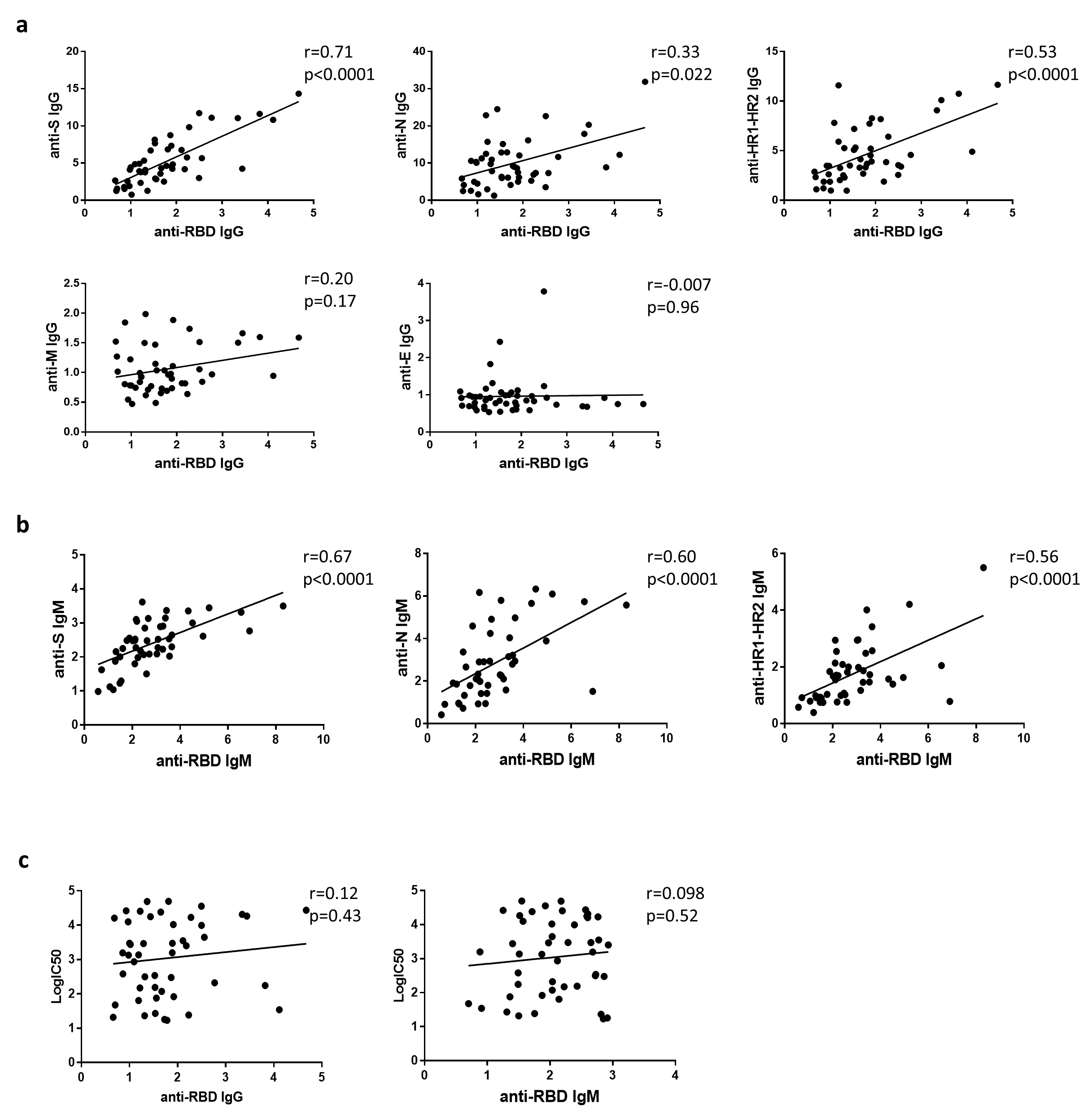


**Supplementary Figure S6. Relationships between anti-RBD antibody and antibodies against other proteins in SARS-CoV-2. a** The correlations of normalized OD450 nm values between the anti-RBD IgG level and the anti-S, HR1-HR2, N, M or E IgG levels of recovered patients were analyzed by Spearman correlation test. **b** The correlations of normalized OD450 nm values between the anti-RBD IgM level and the anti-S, HR1-HR2 or N IgM levels of recovered patients were analyzed by Spearman correlation test.

**
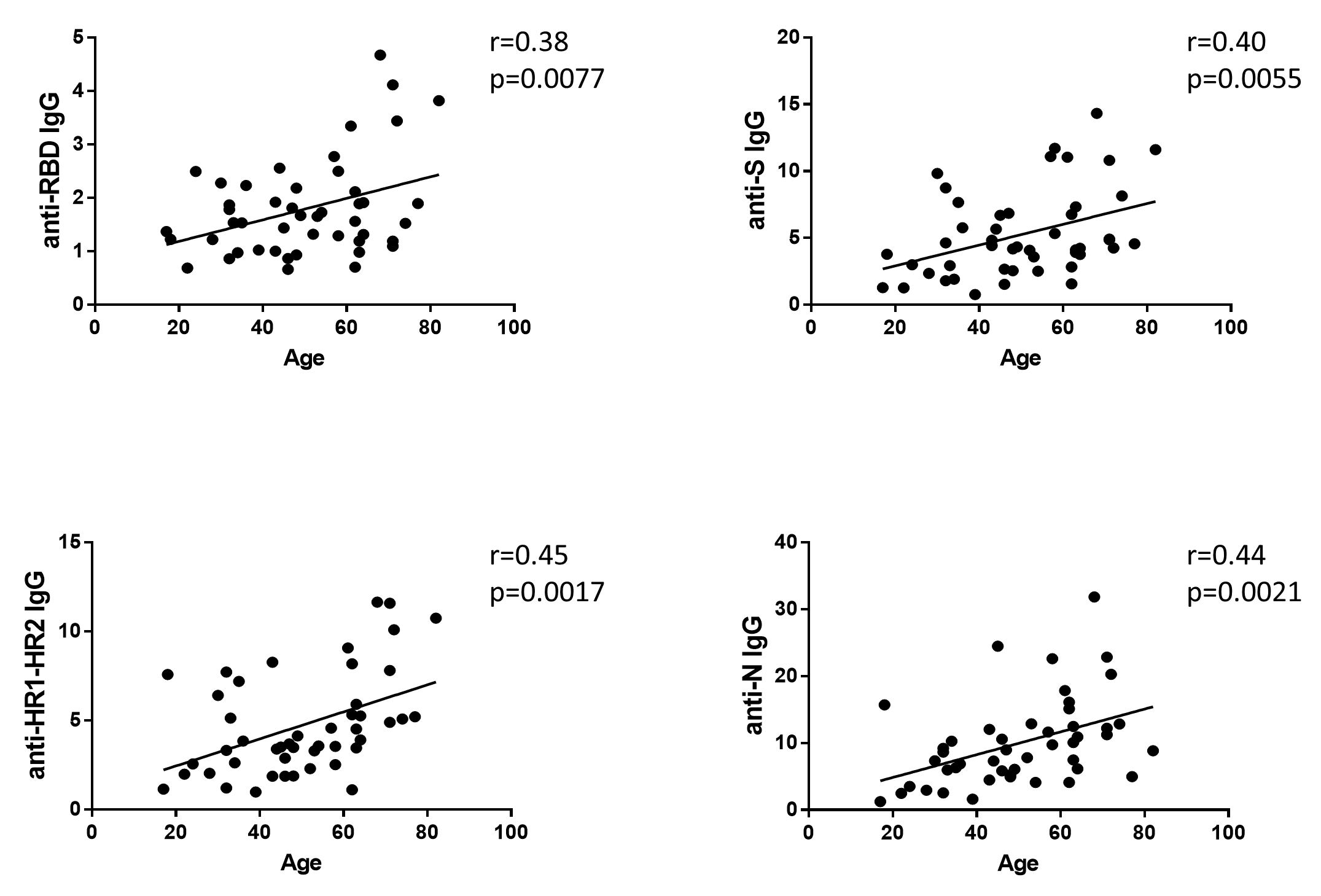
**

**Supplementary Figure S7.** The relationships between ages of recovered patients and the reciprocal titers of SARS-CoV-2-specific IgG to RBD, S, HR1-HR2 and N were analyzed by Spearman correlation test.
